# Partial replacement of high-fat diet with *n*-3 PUFAs enhanced beef tallow attenuates dyslipidemia and endoplasmic reticulum stress in tunicamycin-injected rats

**DOI:** 10.3389/fnut.2023.1155436

**Published:** 2023-03-16

**Authors:** Jiaxiang Zheng, Jisu Lee, Jaemin Byun, Daeung Yu, Jung-Heun Ha

**Affiliations:** ^1^Department of Food Science and Nutrition, Dankook University, Cheonan, Republic of Korea; ^2^Center for Discovery and Innovation, Hackensack Meridian Health, Nutley, NJ, United States; ^3^Department of Food and Nutrition, Changwon National University, Changwon, Republic of Korea; ^4^Interdisciplinary Program in Senior Human-Ecology, Major in Food and Nutrition, Changwon National University, Changwon, Republic of Korea; ^5^Research Center for Industrialization of Natural Neutralization, Dankook University, Yongin, Republic of Korea

**Keywords:** high fat high cholesterol diet, beef tallow, *n*-3 PUFAs, dyslipidemia, unfolded protein responses

## Abstract

**Introduction:**

Metabolic syndrome (MetS) is considered as a complex, intertwined multiple risk factors that directly increase the risk of various metabolic diseases, especially cardiovascular atherosclerotic diseases and diabetes mellitus type 2. While lifestyle changes, including dietary intervention are effective in mitigating or preventing MetS, there are no specific therapies against MetS. Typical western diets comprise of high saturated fatty acid, cholesterol, and simple sugar; consequently their consumption may increase the potential pathological developmental risk of MetS. Partial replacement of dietary fatty acids with polyunsaturated fatty acids (PUFAs) is widely recommended measure to manage MetS-related disorders.

**Methods:**

In the present study, we used rat model to investigate the role of *n*-3 PUFA enriched beef tallows (BT) on MetS and tunicamycin (TM)-induced endoplasmic reticulum (ER) stress, by partially replacing dietary fat (lard) with equal amounts of two different BTs; regular BT or *n*-3 PUFA-enriched BT. The experimental rats were randomly assigned to three different dietary groups (*n* = 16 per group): (1) high-fat and high-cholesterol diet (HFCD); (2) HFCD partially replaced with regular BT (HFCD + BT1); (3) HFCD partially replaced with *n*-3 enhanced BT (w/w) (HFCD + BT2). After 10 weeks of dietary intervention, each experimental rodent was intraperitoneally injected with either phosphate-buffered saline or 1 mg/kg body weight of TM.

**Results:**

HFCD + BT2 showed improved dyslipidemia before TM injection, and increased serum high-density lipoprotein cholesterol (HDL-C) levels after TM injection. BT replacement groups had significantly reduced hepatic triglyceride (TG) levels, and decreased total cholesterol (TC) and TG levels in epididymal adipose tissue (EAT). Furthermore, BT replacement remarkably attenuated TM-induced unfolded protein responses (UPRs) in liver, showing reduced ER stress, with BT2 being more effective in the EAT.

**Discussion:**

Therefore, our findings suggest that partially replacing dietary fats with *n*-3 PUFA to lower the ratio of *n*-6/*n*-3 PUFAs is beneficial in preventing pathological features of MetS by alleviating HFCD- and/or TM-induced dyslipidemia and ER stress.

## 1. Introduction

Metabolic syndrome (MetS) is considered as a complex, intertwined multiple risk factors that directly increase the prevalence of metabolic diseases, such as atherosclerotic cardiovascular disease and type 2 diabetes mellitus (T2DM) (1, 2). There are well-recognized potential risk factors for the development of MetS, such as excessive energy intake, lack of physical activity, and concomitant obesity (3); thus MetS is diagnosed based on waist circumference, blood pressure, fasting blood-glucose, serum triglyceride (TG), and high-density lipoprotein cholesterol (HDL-C) levels (4). Globalization and economic development have spurred changes in dietary patterns globally toward western type (5) which contain more fat (saturated fats and *trans* fats), cholesterol, simple sugar, and higher amounts of processed foods. Continuous consumption of western style of diet undermines the prevalence of obesity, diabetes and cardiovascular diseases (CVDs) (6, 7). MetS has become a global health problem, and finding a dietary interventional method may be an effective and preferable strategy for prevention and progression of MetS (8). For instance, the Mediterranean dietary pattern is widely recommended as against western-style diet as it contains lower saturated fatty acids (SFAs) and higher polyunsaturated fatty acids (PUFAs). The benefits of the Mediterranean diet is largely due to the difference in fatty acids (FAs) compositions compared with western diet (9, 10).

Among the abundant PUFAs in the Mediterranean diet, the *n*-3 PUFAs may exert their influence in the prevention of systemic inflammation, and the reduction of the incidence of CVDs, together with delaying the progression of metabolic diseases (11, 12). *n*-6 PUFAs are also known to regulate renal and pulmonary function, vascular tone and inflammatory response (13, 14), but have distinguishable role in metabolic responses compared to *n*-3 PUFAs because of their competitive functions. As for FAs consumption, western diet is typified with the lower consumption of *n*-3 PUFAs, and excessive consumption of *n*-6 PUFAs results in an improper *n*-6/*n*-3 ratio (15). Moreover, the high *n*-6/*n*-3 ratio directly promotes the pathogenesis of multiple diseases, including CVDs, cancer, and autoimmune diseases, while elevated *n*-3 levels reduce the *n*-6/*n*-3 ratio exerting inhibitory effects in these diseases (16, 17). The recommended range of dietary fatty acid intake as per the American Heart Association (AHA) for adult is approximately 25–35 energy percentage (E%), while that for the PUFAs is approximately 5–10 E% (18).

The endoplasmic reticulum (ER) is an important cellular organelles, where newly synthesized proteins are folded, and subsequently subjected to post-translational modifications (19). Alterations in the function of the ER can result in accumulation of unfolded or misfolded proteins resulting in disruption of the normal ER homeostasis, the condition referred to as ER stress (20). ER stress can trigger the activation of the unfolded protein responses (UPRs), the adaptive mechanism that either corrects the misfolded proteins, or induces cellular apoptosis (21). ER stress-triggered UPRs, and apoptotic responses are closely associated with the pathophysiology of various abnormal MetS or diseases, such as obesity, diabetes, dyslipidemia and atherosclerosis (22). Moreover, the challenges of translational abnormal homeostasis on lipid metabolism caused by chronic ER stress are key risk factors in the pathogenesis of MetS, including T2DM, obesity and dyslipidemia (23, 24).

Beef tallow (BT) is a by-product of beef processing, and is extensively used in the food industry because of its high thermal and oxidative stability (25). The unique and desirable flavor of BT after baking, makes it popular as a food supplement in frying, bakery products, and margarine production (26). Beef is a major component of western diet, and BT can be naturally consumed when eating beef (27). Beef is a nutrient-dense resource, possessing variety of nutrients, such as essential amino acids, B vitamins, minerals (iron, zinc, and selenium), and various FAs (28). However, beef consumption may also induce some risks to health, due to innate characteristics to have high cholesterol and SFAs, low PUFAs, and/or inappropriate ratio of *n*-6/*n*-3 PUFA (29). Interestingly, *n*-3 enriched linseed increases the content of *n*-3 in cattle muscle and adipose tissue, resulting in a lower ratio of *n*-6/*n*-3 (30). Indeed, reducing the intake of SFAs and increasing the intake of *n*-3 PUFAs when consuming beef may be beneficial to health (31). Clinical data also support the beneficial effects of replacing SFAs with PUFAs, in reducing the risk of MetS (32). Therefore, replacing dietary fats from typical western diet, with *n*-3 PUFA-enhanced BT can be a good strategy to manage MetS.

We hypothesize that partial replacement of dietary fat with PUFAs could be an effective and reasonable strategy to reduce or prevent the incidence of MetS. Diets containing a lower ratio of *n*-6/*n*-3 PUFAs appear to be more beneficial in mitigating the development of MetS; this can be applicable by adjusting to a reasonable balance, the intake of *n*-6 and *n*-3 (33, 34). To this end, we partially replaced the fatty acids in high-fat and high-cholesterol diet (HFCD) with either regular BT or a *n*-3 enhanced BT, to investigate whether a dietary intervention could alleviate the MetS induced by the HFCD diet. Although, BTs contains relatively higher levels of SFAs, we increased the *n*-3 amount in BT by adding *n*-3 enriched *perilla* pomace to cattle feed. Therefore, in this study, we aimed to investigate whether partial replacement of dietary fat with *n*-3 enhanced BTs could attenuate or prevent the risk factors of HFCD-inducible MetS and tunicamycin (TM)-induced ER stress in Sprague-Dawley (SD) rats.

## 2. Materials and methods

### 2.1. Animal experiments

All animal studies were conducted in accordance with the procedures of the Institutional Animal Care Use Committee of Dankook University (IACUC, No. DKU-21-051). Five-week-old male SD rats were obtained from Doo Yeol Biotech, Inc. (Seoul, Korea). The SD rats were housed under controlled conditions of room temperature 20 ± 2°C and 50–55% relative humidity with a 12 h light/12 h dark cycle, and *ad libitum* access to water. The experimental SD rats were randomly assigned to three groups (*n* = 16 per group) based on type of dietary intervention: (1) high-fat and high-cholesterol diet (HFCD); (2) lard of HFCD partially replaced with 11% regular beef tallow (w/w) (HFCD + BT1); (3) lard of HFCD partially replaced with 11% beef tallow (w/w) containing a lower *n-6*/*n-3* ratio (HFCD + BT2). The amount of energy obtained from fat was 46.18% in all the diet types. The composition of diets and fatty acid are shown in Tables 1, 2, respectively. BT (Greengrass Bio, Chungju, Korea) was obtained from retroperitoneal adipose tissue (RAT) of either black Angus fed either regular diet (BT1) or *perilla* pomace (BT2). After 10 weeks of experimental diet feeding, each group was further divided into two subgroups (*n* = 8 per group): (1) intraperitoneal injection of phosphate-buffered saline (PBS) or (2) intraperitoneal injection of TM (1 mg/kg body weight). Following the injection of either PBS or TM, the experimental rats were fasted. Twelve hours after PBS or TM injection, the rats were humanely euthanized by thoracotomy after CO_2_ narcosis. Whole blood was collected by cardiac puncture, and the serum was separated by centrifugation (3,000 × *g* at 4°C for 15 min). The liver and the white adipose tissues (WATs), including epididymal, mesenteric, retroperitoneal and perirenal tissues, were collected and weighed, subsequently kept at −80°C until analysis.

**TABLE 1:** Composition of the experimental diets.

Diet ingredient (g)	HFCD	HFCD + BT1	HFCD + BT2
Casein	220	220	220
L-cysteine	3.4	3.4	3.4
Sucrose	100	100	100
Cornstarch	147.766	147.766	147.766
Dextrose	155	155	155
t-butylhydroquinone	0.034	0.034	0.034
Cellulose	58	58	58
Mineral mix^1^	43	43	43
Vitamin mix^2^	19	19	19
Choline bitartrate	2.8	2.8	2.8
Soybean oil	70	70	70
Lard	166	56	56
Cholesterol	10	10	10
Cholic acid	5	5	5
Beef tallow 1	–	110	–
Beef tallow 2	–	–	110
Total	1,000	1,000	1,000
Energy (kcal/g)	4.79	4.79	4.79
Fat (kcal%)	46.18	46.18	46.18

^1^AIN-93-GX mineral mixture, ^2^AIN-93-VX vitamin mixture. HFCD, high-fat and high-cholesterol diet; HFCD + BT1, high-fat and high-cholesterol diet + regular beef tallow; HFCD + BT2, high-fat and high-cholesterol diet + beef tallow containing a lower *n*-6/*n*-3 ratio.

**TABLE 2:** Fatty acid composition of beef tallow.

Fatty acids (%)	Diet	
	BT1	BT2
*n*-6	1.211	1.453
*n*-3	1.343	3.585
*n*-6/*n*-3	0.9	0.4

BT, beef tallow; *n*-3, omega-3 fatty acids; *n*-6, omega-6 fatty acids.

### 2.2. Dietary fatty acid analysis

The fatty acid composition of the experimental diets was measured by the methyl esterification of boron trifluoride (BF3)-methanol following previously published methods (33–36). Gas chromatography (GC) (Agilent Technologies 6890N, Agilent Technologies, CA, USA) was employed for analyzing the FAs, and the results were assessed by comparison with a standard FA reference (Sigma-Aldrich Co., St. Louis, MO, USA). The FA composition of the experimental diets is presented in Table 3.

**TABLE 3:** Fatty acid composition of the experimental diets.

Fatty acid (% total fatty acids)	HFCD	HFCD + BT1	HFCD + BT2
C_12:0_, Lauric acid	ND	0.42 ± 0.02^a^	0.37 ± 0.02^b^
C_14:0_, Myristic acid	1.49 ± 0.07^c^	2.78 ± 0.14^b^	3.41 ± 0.17^a^
C_15:0_, Pentadecanoic acid	ND	0.64 ± 0.03^b^	0.72 ± 0.04^a^
C_16:0_, Palmitic acid	19.97 ± 1.00^a^	18.95 ± 0.95^a^	20.13 ± 1.01^a^
C_17:0_, Heptadecanoic acid	3.11 ± 0.16^b^	11.72 ± 0.59^a^	10.62 ± 0.53^a^
C_18:0_, Stearic acid	12.23 ± 0.61^a^	10.31 ± 0.52^b^	12.46 ± 0.62^a^
C_14:1_, Myristoleic acid	ND	1.18 ± 0.06^a^	1.07 ± 0.05^a^
C_16:1_, Palmitoleic acid	1.81 ± 0.09^b^	3.70 ± 0.19^a^	3.56 ± 0.18^a^
C_18:1_, Oleic acid	30.79 ± 1.54^a^	24.43 ± 1.22^b^	23.25 ± 1.16^b^
C_20:1_, Eicosenoic acid	1.21 ± 0.06^b^	1.45 ± 0.07^a^	1.60 ± 0.08^a^
C_18:2_, Linoleic acid	24.81 ± 1.24^a^	18.33 ± 0.92^b^	16.11 ± 0.81^b^
C_20:2_, Eicosadienoic acid	ND	0.42 ± 0.02^b^	0.52 ± 0.03^a^
C_18:3_, Linolenic acid	4.59 ± 0.23^b^	5.09 ± 0.25^b^	5.88 ± 0.29^a^
C_18:3_, γ-Linolenic acid	ND	0.22 ± 0.01^a^	ND
C_20:3_, Eicosatrienoic acid	ND	0.37 ± 0.02^a^	0.31 ± 0.02^b^
SFAs	36.79 ± 1.84^b^	44.82 ± 2.24^a^	47.70 ± 2.39^a^
MUFAs	33.81 ± 1.69^a^	30.76 ± 1.54^ab^	29.49 ± 1.47^b^
PUFAs	29.40 ± 1.47^a^	24.42 ± 1.22^b^	22.81 ± 1.14^b^
*n*-6	24.81 ± 1.24^a^	18.55 ± 0.93^b^	16.11 ± 0.81^b^
*n*-3	4.59 ± 0.23^c^	5.45 ± 0.27^b^	6.18 ± 0.31^a^
*n*-6/*n*-3	5.41 ± 0.00^a^	3.40 ± 0.00^b^	2.60 ± 0.00^c^
*n*-9	30.79 ± 1.54^a^	24.43 ± 1.22^b^	23.25 ± 1.16^b^

HFCD, high-fat and high-cholesterol diet; HFCD + BT1, high-fat and high-cholesterol diet + regular beef tallow; HFCD + BT2, high-fat and high-cholesterol diet + beef tallow containing a lower *n*-6/*n*-3 ratio; ND, not detected; SFAs, saturated fatty acids; MUFAs, monounsaturated fatty acids; PUFAs, polyunsaturated fatty acids; *n*-3, omega-3 fatty acids; *n*-6, omega-6 fatty acids; *n*-9, omega-9 fatty acids. Values are presented as mean ± standard deviation (*n* = 3 per group). Values with different letters are significantly different using one-way ANOVA followed by a *post hoc* test with Tukey’s multiple comparison method, *p* < 0.05. Unit is presented as a percentage (%) of total fatty acids.

### 2.3. Serum lipid and hepatic biological function panel

Serum levels of TG, total cholesterol (TC), and HDL-C were measured using commercial kits (TG Assay Kit, TC Assay Kit, and HDL-C Assay Kit, Embiel, Gunpo, Korea). The non-HDL-C level was calculated by subtracting TG and HDL-C values from the TC levels (37). The cardiac risk factor (CRF) was calculated by using the formula: CRF = TC/HDL-C (38, 39). Indices for hepatic biological function were determined by measuring serum aspartate aminotransferase (AST), alanine transaminase (ALT), and alkaline phosphatase (ALP) using commercial kits (AST Assay Kit, ALT Assay Kit, and ALP Assay Kit, Embiel). The detailed methods have been previously described (33–36).

### 2.4. Serum glucose and insulin levels

Fasting glucose (Crystal Chem, Downers Grove, IL, USA), and insulin (Mercodia AB, Uppsala, Sweden) levels at the final sacrifice were measured using standard commercial kits according to the manufacturer’s instructions. The homeostatic index of insulin resistance (HOMA-IR) values was calculated by the following established formula: HOMA-IR = serum insulin (μU/L) × serum glucose (mg/dL)/405 (40).

### 2.5. Lipid contents in the liver and epididymal adipose tissue

Lipids were extracted from the liver and epididymal adipose tissues (EAT) by the previously described method of Bligh and Dyer (41), which has been used in similar experimental settings earlier (33–36).

### 2.6. Analysis of the fatty acid composition of whole blood

The methods used for the analysis of the FAs composition of whole blood have been previously described (33–36). A drop of whole blood from each mouse was spiked on a blood spot card (OmegaQuant Analytics, Sioux Falls, SD, USA) pre-coated with an antioxidant mixture. The blood fatty acid composition was analyzed using GC (Agilent Technologies 6890N, Agilent Technologies, CA, USA) as described by Harris and Polreis (42). The fatty acid composition of the whole blood is expressed as a percentage from the total identified FAs as shown in Table 4.

**TABLE 4:** Fatty acid composition of whole blood in rats after tunicamycin challenge.

(% Total fatty acids)	PBS			TM		
	HFCD	HFCD + BT1	HFCD + BT2	HFCD	HFCD + BT1	HFCD + BT2
***n*** **-3 PUFA**						
α-Linolenic acid	0.110 ± 0.072	0.127 ± 0.108	0.127 ± 0.085	0.123 ± 0.075	0.057 ± 0.038	0.063 ± 0.015
Eicosapentaenoic acid (EPA)	0.200 ± 0.066	0.167 ± 0.038	0.303 ± 0.295	0.243 ± 0.137	0.363 ± 0.253	0.157 ± 0.074
Docosapentaenoic acid (DPA)	0.927 ± 0.084	0.920 ± 0.101	0.887 ± 0.178	1.183 ± 0.090	1.243 ± 0.388	1.153 ± 0.159
Docosahexaenoic acid (DHA)	1.170 ± 0.249	1.283 ± 0.407	1.013 ± 0.402	0.947 ± 0.194	1.237 ± 0.348	0.960 ± 0.171
***n*** **-6 PUFA**						
Linoleic acid	13.470 ± 0.493^a^	11.17 ± 1.115^b^	12.57 ± 0.289^ab^	12.10 ± 0.854^ab^	11.77 ± 0.862^ab^	11.50 ± 0.608^ab^
γ-Linolenic acid	0.063 ± 0.023	0.027 ± 0.015	0.030 ± 0.030	0.067 ± 0.040	0.043 ± 0.032	0.020 ± 0.010
Eicosadienoic acid	0.290 ± 0.141	0.273 ± 0.040	0.327 ± 0.035	0.413 ± 0.040	0.213 ± 0.035	0.293 ± 0.101
Dihomo-γ-linolenic acid	1.067 ± 0.067	0.910 ± 0.108	1.110 ± 0.115	0.870 ± 0.171	0.980 ± 0.212	0.903 ± 0.045
Arachidonic acid	21.67 ± 1.159	20.77 ± 0.651	19.97 ± 1.484	22.70 ± 0.400	22.50 ± 1.200	22.20 ± 1.217
Docosatetraenoic acid	1.397 ± 0.261	1.223 ± 0.058	1.233 ± 0.038	1.597 ± 0.072	1.240 ± 0.104	1.407 ± 0.205
Docosapentaenoic acid	0.227 ± 0.049	0.250 ± 0.095	0.293 ± 0.144	0.173 ± 0.075	0.220 ± 0.010	0.217 ± 0.083
***n*** **-9 PUFA**						
Oleic acid	14.10 ± 2.100	18.23 ± 3.066	17.63 ± 2.641	13.47 ± 0.321	14.67 ± 0.929	14.57 ± 0.808
Eicosenoic acid	0.180 ± 0.010^ab^	0.270 ± 0.056^a^	0.210 ± 0.026^ab^	0.203 ± 0.031^ab^	0.093 ± 0.040^b^	0.157 ± 0.127^ab^
Nervonic acid	0.160 ± 0.062	0.063 ± 0.075	0.177 ± 0.021	0.220 ± 0.069	0.247 ± 0.076	0.203 ± 0.127
**SFA**						
Palmitoleic acid	0.677 ± 0.234	1.510 ± 0.553	1.120 ± 0.524	0.603 ± 0.196	0.600 ± 0.130	0.790 ± 0.177
Myristic acid	0.157 ± 0.010	0.377 ± 0.195	0.243 ± 0.038	0.193 ± 0.012	0.203 ± 0.055	0.360 ± 0.120
Palmitic acid	27.07 ± 0.723^ab^	26.43 ± 0.709^b^	26.73 ± 1.079^ab^	27.93 ± 0.551^ab^	27.60 ± 0.100^ab^	28.37 ± 0.351^a^
Stearic acid	16.40 ± 0.781	15.00 ± 1.058	15.23 ± 0.987	16.33 ± 0.321	15.90 ± 0.346	15.67 ± 1.007
Lignoceric acid	0.070 ± 0.017	0.097 ± 0.015	0.103 ± 0.060	0.083 ± 0.006	0.080 ± 0.036	0.110 ± 0.061
***Trans*** **FA**						
*Trans* palmitoleic acid	0.170 ± 0.026^ab^	0.240 ± 0.053^ab^	0.233 ± 0.071^ab^	0.133 ± 0.047^b^	0.153 ± 0.072^b^	0.310 ± 0.030^a^
*Trans* oleic acid	0.080 ± 0.052^c^	0.323 ± 0.070^a^	0.197 ± 0.015^abc^	0.097 ± 0.045^bc^	0.270 ± 0.010^a^	0.220 ± 0.070^ab^
*Trans* linoleic acid	0.010 ± 0.000	0.010 ± 0.000	0.010 ± 0.000	0.010 ± 0.000	0.010 ± 0.000	0.010 ± 0.000
*Trans* linoleic acid 2	0.040 ± 0.044	0.037 ± 0.015	0.050 ± 0.010	0.020 ± 0.000	0.023 ± 0.015	0.040 ± 0.000
*Trans* linoleic acid 3	0.047 ± 0.046	0.057 ± 0.015	0.033 ± 0.006	0.047 ± 0.021	0.053 ± 0.031	0.063 ± 0.012
*n*-3	2.407 ± 0.180	2.497 ± 0.489	2.330 ± 0.095	2.497 ± 0.202	2.900 ± 0.210	2.333 ± 0.167
*n*-6	38.18 ± 1.078^a^	34.62 ± 1.697^b^	35.53 ± 1.324^ab^	37.92 ± 0.666^a^	36.96 ± 0.976^a^	36.54 ± 0.872^a^
*n*-9	14.44 ± 2.150	18.57 ± 3.082	18.02 ± 2.681	13.89 ± 0.334	15.01 ± 0.977	14.93 ± 0.846
SFAs	43.69 ± 1.345^a^	41.91 ± 1.806^a^	42.31 ± 2.142^a^	44.54 ± 0.575^a^	43.78 ± 0.377^ab^	44.50 ± 0.947^ab^
*Trans* FAs	0.347 ± 0.119^bc^	0.667 ± 0.057^a^	0.523 ± 0.075^ab^	0.307 ± 0.067^c^	0.510 ± 0.044^abc^	0.643 ± 0.083^a^
MUFAs	15.12 ± 2.381	20.08 ± 3.537	19.14 ± 3.205	14.49 ± 0.526	15.61 ± 1.017	15.72 ± 0.957
*n*-6/*n*-3	15.94 ± 1.518	14.16 ± 2.372	15.25 ± 0.287	15.24 ± 0.988	12.81 ± 1.232	15.71 ± 0.986
AA/EPA	116.3 ± 37.20	128.6 ± 27.30	174.4 ± 92.71	117.6 ± 68.81	84.31 ± 52.61	163.5 ± 73.01

PBS, phosphate-buffered saline; TM, tunicamycin; HFCD, high-fat and high-cholesterol diet; HFCD + BT1: high-fat and high-cholesterol diet + regular beef tallow; HFCD + BT2: high-fat and high-cholesterol diet + beef tallow containing a lower ratio of *n*-6/*n*-3; *n*-3, omega-3 fatty acids; *n*-6, omega-6 fatty acids; *n*-9, omega-9 fatty acids; SFAs, saturated fatty acids; *Trans* FAs, *trans* fatty acid; MUFAs, monounsaturated fatty acids; AA, arachidonic acid; EPA, eicosapentaenoic acid. Values are presented as the mean ± standard deviation (*n* = 3 per group). Data are analyzed by two-way ANOVA followed by Tukey’s multiple comparisons test to determine the interactions or the main effects (diet and TM stimulation). Mean values labeled with different letters indicate statistically significant difference (*p* < 0.05).

### 2.7. Western blot analysis

The proteins from the liver and EAT were extracted using ice-cold radio-immunoprecipitation assay buffer (RIPA) lysis buffer (ATTO, Tokyo, Japan) containing protease inhibitors and phosphatase inhibitors (Thermo Fisher Scientific, Waltham, MA, USA). The proteins were separated using 12% sodium dodecyl sulfate-polyacrylamide gel electrophoresis (SDS-PAGE), and transferred to polyvinylidene fluoride (PVDF) membranes (Bio-Rad Laboratories, Hercules, CA, USA) at 120 volts for 90 min. The membrane was blocked with 5% skim milk (BD Difco™, Franklin Lakes, NJ, USA) for 1 h, and subsequently immunoblotted with relevant primary antibodies at 4°C overnight. The membranes were probed with a secondary antibodies for 1 h, and reacted with SuperSignal Chemiluminescent Substrate (Thermo Fisher Scientific). Chemiluminescence was detected using a Danvich Chemi Fluoro Imager (Danvich-K, Seoul, Korea). Visualization of the detected proteins were analyzed using ImageJ software (v.1.8 National Institutes of Health, Bethesda, MD, USA), with GAPDH as an internal control (34). Details of antibodies used for western blot analysis is listed in Table 5.

**TABLE 5:** List of antibodies for western blot analysis.

Antibody	Manufacturer	Catalog number	Dilution
BiP	Cell signaling technology (CST, Danvers, MA, USA)	3,183	1:1000
XBP-1	CST	40,435	1:1000
ATF4	CST	11,815	1:1000
CHOP	CST	2,895	1:1000
GAPDH	Santa Cruz, Dallas, TX, USA	sc-47724	1:1000
Anti-rabbit IgG	CST	7,074	1:3000
Anti-mouse IgG	CST	7,076	1:1000

BiP, binding immunoglobulin protein; XBP-1, X-box binding protein; ATF4, activating transcription factor 4; CHOP, C/EBP homologous protein; GAPDH, glyceraldehyde-3-phosphate dehydrogenase.

### 2.8. Statistical analysis

Data are expressed as mean ± standard deviations (SDs), or as Box-and-whisker plots. Box-and-whisker plots indicated the average of the assigned group with a “+” sign, and depicted the minimum, the lower (25th percentile), the median (50th percentile), the upper (75th percentile), and the maximum ranked samples. One-way analysis of variance (ANOVA) was employed to compare the effects of the dietary intervention. The interaction of diet and TM stimulation was analyzed using two-way ANOVA with Tukey’s *post hoc* test. Statistical significance was set at *p* < 0.05. Statistical analyses were performed using XLSTAT 2019 (Addinsoft Inc., Paris, France) or SPSS 26.0 (Statistical Package for Social Science, IBM Corp., Armonk, NY, USA), and all figures are depicted using GraphPad prism 5 (GraphPad Software Inc., San Diego, CA, USA). Table 6 presents a summary of the two-way ANOVA for the main effects of dietary intervention and TM-injection, and their interaction.

**TABLE 6:** Statistical summary.

Parameter	Factor. *P*-values		
	Tunicamycin	Diet	Tunicamycin X diet
**Serum glucose, insulin levels**			
Glucose	ns *p* = 0.194	ns *p* = 0.319	ns *p* = 0.791
Insulin	**p < 0.01	ns *p* = 0.859	ns *p* = 0.950
HOMA-IR	ns *p* = 0.079	ns *p* = 0.292	ns *p* = 0.315
**Relative tissue weights**			
Liver	ns *p* = 0.165	ns *p* = 0.996	ns *p* = 0.965
EAT	**p < 0.01	ns *p* = 0.219	ns *p* = 0.972
MAT	*p < 0.05	ns *p* = 0.123	ns *p* = 0.271
RAT	***p < 0.001	ns *p* = 0.206	ns *p* = 0.340
PAT	***p < 0.001	ns *p* = 0.912	ns *p* = 0.202
WAT	***p < 0.001	ns *p* = 0.189	ns *p* = 0.547
**Serum lipid profiles**			
Total cholesterol	****p < 0.0001	****p < 0.0001	***p < 0.001
HDL-cholesterol	****p < 0.0001	****p < 0.0001	*p < 0.05
Non-HDL-cholesterol	****p < 0.0001	****p < 0.0001	***p < 0.001
Triglyceride	****p < 0.0001	****p < 0.0001	**p < 0.01
Cardiac risk factor	****p < 0.0001	****p < 0.0001	****p < 0.0001
**Lipid contents in the liver tissue**			
Hepatic TG	****p < 0.0001	****p < 0.0001	**p < 0.01
Hepatic TC	ns *p* = 0.152	**p < 0.01	ns *p* = 0.257
**Lipid contents in the epididymal adipose tissue**			
EAT TG	***p < 0.0001	****p < 0.0001	****p < 0.0001
EAT TC	**p < 0.01	****p < 0.0001	ns *p* = 0.193
**Hepatic function parameters**			
Serum AST	****p < 0.0001	****p < 0.0001	***p < 0.001
Serum ALT	****p < 0.0001	****p < 0.0001	****p < 0.0001
Serum ALP	****p < 0.0001	****p < 0.0001	ns *p* = 0.417

*p < 0.05, **p < 0.01, ***p < 0.001, ****p < 0.0001; ns, not significant. HOMA-IR, homeostasis model assessment of insulin resistance; EAT, epididymal adipose tissue; MAT, mesenteric adipose tissue; RAT, retroperitoneal adipose tissue; PAT, perirenal adipose tissue; WAT, white adipose tissue; HDL, high-density lipoprotein; TG, triglyceride; TC, total cholesterol; AST, aspartate aminotransferase; ALT, alanine aminotransferase; ALP, alkaline phosphatase.

## 3. Results

### 3.1. Effects of partial replacement of dietary fat with BTs on body weight change, daily food intake, daily energy intake, and food efficiency ratio

**FIGURE 1:**
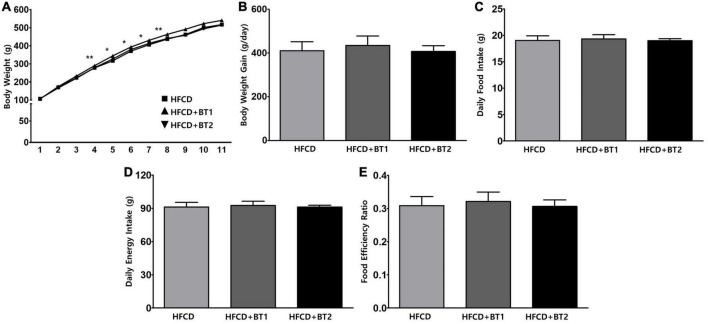
Effects of partial replacement of dietary fat with beef tallows (BTs) on the body weight change, daily food intake, daily energy intake, and food efficiency ratio in Sprague Dawley rats. Five-week-old male Sprague Dawley rats were fed either a high-fat and high-cholesterol (HFCD), lard of HFCD partially replaced with regular beef tallow (HFCD + BT1), or lard of HFCD partially replaced with BT containing a lower *n*-6/*n*-3 ratio (HFCD + BT2) diets, respectively for 10 weeks. **(A)** Body weight changes, **(B)** daily body weight gain (final body weight – initial body weight), **(C)** daily food intake, **(D)** daily energy intake, and **(E)** food efficiency ratio (FER) was measured. Values are presented as means ± SD; *n* = 16 per individual group. Data were analyzed using one-way ANOVA followed by Tukey’s multiple comparisons test. * and ** denotes a significant main effect of diet at *p* < 0.05 and *p* < 0.01, respectively. HFCD, high-fat and high-cholesterol; HFCD + BT1, high-fat and high-cholesterol diet + regular beef tallow; HFCD + BT2, high-fat and high-cholesterol diet + BT containing a lower *n*-6/*n*-3 ratio.

Experimental SD rats were fed HFCD, HFCD with BT1, or HFCD with BT2 for 10 weeks (*n* = 16 per group). After 4 weeks of dietary intervention, the HFCD + BT1 group had significantly higher body weight (BW) than HFCD and HFCD + BT2 groups, but the data for BW in the last 3 weeks of the intervention was not statistically significant (Figure 1A). High-fat and high-cholesterol diets have been reported to increase BW in rats (43). Though we found no significant difference in daily BW gain between the three groups (Figure 1B). Daily food intake was calculated by dividing the total dietary intake by the experimental period, and there was no significant difference among the three groups (Figure 1C). Daily energy intake, and food efficiency ratio (FER) did not substantially change with dietary intervention (Figures 1D, E). Therefore, in our experimental setting, partial replacement of dietary fat in HFCD with BTs did not significantly affect the daily food intake, daily energy intake and FER.

### 3.2. Effects of partial replacement of dietary fat with BTs on serum glucose and insulin levels

**FIGURE 2:**
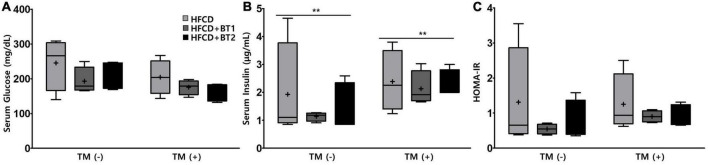
Effects of partial replacement of dietary fat with beef tallows (BTs) and tunicamycin (TM) challenge on the serum glucose and insulin levels in Sprague Dawley rats. Five-week-old male Sprague-Dawley rats were fed either a high-fat and high-cholesterol diet (HFCD), lard of HFCD partially replaced with regular beef tallow (HFCD + BT1), or lard of HFCD partially replaced with BT containing a lower *n*-6/*n*-3 ratio (HFCD + BT2) for 10 weeks, followed by injection with phosphate-buffered saline (PBS), or TM (1 mg/kg). **(A)** Serum glucose level, **(B)** serum insulin level, and **(C)** homeostasis model assessment of insulin resistance (HOMA-IR) level. Data were analyzed using two-way ANOVA followed by Tukey’s multiple comparisons test to determine the interactions or the main effects (diet and TM stimulation). Values are presented as a Box-and-Whisker plots representing 4 rats per individual group. **Denotes a significant main effect of TM stimulation at *p* < 0.01. The mean values are indicated by “+” sign. HFCD, high-fat and high-cholesterol; HFCD + BT1, high-fat and high-cholesterol diet + regular beef tallow; HFCD + BT2, high-fat and high-cholesterol diet + BT containing a lower *n*-6/*n*-3 ratio; TM, tunicamycin; PBS, phosphate-buffered saline.

We further investigated whether partial replacement of dietary fat with BT alters the serum glucose, insulin, and HOMA-IR at the end of dietary intervention. IR is characterized by an increased blood glucose, and excessive insulin secretion is necessary due to decreased insulin sensitivity (44), while a reduction in HOMA-IR is often considered to be an evidence for improved insulin sensitivity (45). TM is considered to reduce insulin-provoked glucose transport and thus cause an increase in the levels of serum glucose (46). Serum glucose levels in rats fed with HFCD + BT1 and HFCD + BT2 in the presence or absence of TM were lower than those in HFCD group, although the differences were not statistically significant (Figure 2A). TM administration increased serum insulin levels compared to PBS treatment group (*p* < 0.01; Figure 2B). Additionally, after TM-injection, both the HFCD + BT1 and HFCD + BT2 groups showed a relatively lower HOMA-IR level of 72.2 and 74.4%, respectively, compared to the HFCD group (considered as 100%), but there was no statistical differences (Figure 2C). Fasting serum insulin levels, and HOMA-IR in rats fed with HFCD + BT1 in the absence of TM exhibited lower trends than in HFCD and HFCD + BT2 groups, although there was no statistical differences (Figures 2B, C). These results suggest that partial replacement of dietary fat with BT1 may enhance insulin sensitivity.

### 3.3. Effects of partial replacement of dietary fat with BTs on liver and adipose tissue weights

**FIGURE 3:**
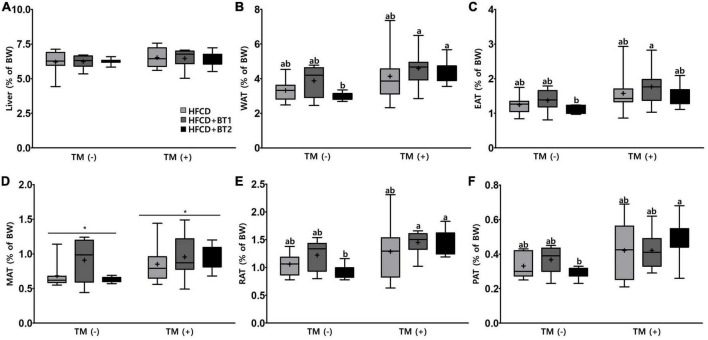
Effects of partial replacement of dietary fat with beef tallows (BTs) and tunicamycin (TM) challenge on liver and adipose tissue weights in Sprague Dawley rats. Five-week-old male Sprague Dawley rats were fed either a high-fat and high-cholesterol (HFCD), lard of HFCD partially replaced with regular beef tallow (HFCD + BT1), or lard of HFCD partially replaced with BT containing a lower *n*-6/*n*-3 ratio (HFCD + BT2) diets, respectively for 10 weeks, and then treated with PBS or TM (1 mg/kg) (*n* = 8 per group). **(A)** Liver weight, **(B)** white adipose tissue (WAT) weight, **(C)** epididymal adipose tissue (EAT) weight, **(D)** mesenteric adipose tissue (MAT) weight, **(E)** retroperitoneal adipose tissue (RAT) weight, **(F)** perirenal adipose tissue (PAT) weight were measured. Values are presented as Box-and-Whisker plots representing eight rats per group. The mean values are indicated by “+” sign. Data were analyzed using two-way ANOVA followed by Tukey’s multiple comparisons test to determine the interactions or the main effects (diet and TM stimulation). Asterisk indicates a significant effect for TM stimulation (*p < 0.05). Mean values labeled with different letters indicate statistically significant difference, *p* < 0.05. HFCD, high-fat and high-cholesterol; HFCD + BT1, high-fat and high-cholesterol diet + regular beef tallow; HFCD + BT2, high-fat and high-cholesterol diet + BT containing a lower *n*-6/*n*-3 ratio; TM, tunicamycin; PBS, phosphate-buffered saline.

To determine whether partial replacement of dietary fat with BTs could attenuate the lipid accumulation in the liver or WATs, the weights of liver and WATs the sum of the weight of [epididymal (EAT), mesenteric (MAT), retroperitoneal (RAT), and perirenal adipose tissues (PAT)] were weighed. TM is a pharmacological chemical inducer of ER stress that inhibits N-linked glycosylation of nascent proteins, resulting in the activation of UPRs in mammalian cells (47). No significant difference in the liver weight was observed with presence or absence of TM administration, and dietary intervention (Figure 3A). After TM injection, rats fed HFCD + BT2 had elevated weights of RAT and PAT compared to the PBS-injected group, resulting in elevated weights of total WATs (Figures 3B, E, F). Furthermore, in the TM-injected group, slightly increased MAT compared to the PBS-injected group (*p* < 0.05; Figure 3D) was observed. However, dietary intervention did not cause obvious differences in adipose tissue weight among the three groups in the presence or absence of TM administration. These data showed that TM treatment caused accumulation of WAT, while partial replacement of dietary fat with BT2 may be associated with reduced WAT accumulation.

### 3.4. Effects of partial replacement of dietary fat with BTs on serum lipid profiles and cardiovascular parameters

**FIGURE 4:**
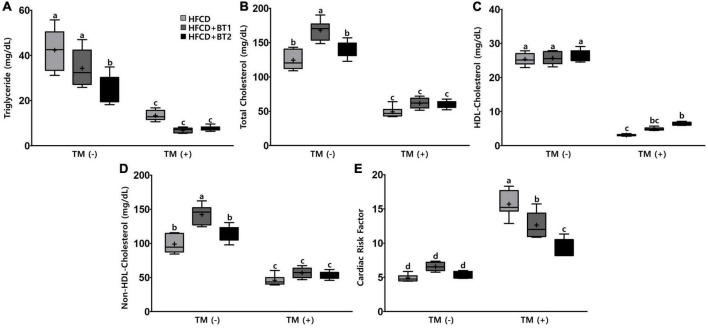
Effects of partial replacement of dietary fat with beef tallows (BTs) and tunicamycin (TM) challenge on serum lipid profiles in Sprague Dawley rats. Five-week-old male Sprague Dawley rats were fed either a high-fat and high-cholesterol (HFCD), lard of HFCD partially replaced with regular beef tallow (HFCD + BT1), or lard of HFCD partially replaced with BT containing a lower *n*-6/*n*-3 ratio (HFCD + BT2) diets, respectively for 10 weeks, and then treated with PBS or TM (1 mg/kg). **(A)** Serum triglyceride levels, **(B)** serum total cholesterol levels, **(C)** high-density lipoprotein (HDL)-cholesterol levels, **(D)** non-HDL cholesterol levels, **(E)** cardiac risk factor (CRF) was measured. Values are presented as Box-and-Whisker plots representing eight rats per group. The mean values are indicated by “+” sign. Data were analyzed using two-way ANOVA followed by Tukey’s multiple comparisons test to determine the interactions or the main effects (diet and TM stimulation). Mean values labeled with different letters indicate statistically significant difference, *p* < 0.05. HFCD, high-fat and high-cholesterol; HFCD + BT1, high-fat and high-cholesterol diet + regular beef tallow; HFCD + BT2, high-fat and high-cholesterol diet + BT containing a lower *n*-6/*n*-3 ratio; TM, tunicamycin; PBS, phosphate-buffered saline.

To investigate the effects of partial replacement of dietary fat with BTs on lipid-lowering and CVD risk factors, we measured the CVD-related serum lipid panels, and calculated the CRF. After TM administration, serum TG, TC, HDL-C, and non-HDL-C levels decreased by 72.27, 60.57, 81.30, and 56.04%, respectively, whereas CRF levels were significantly increased by 124.44% (Figure 4). Serum TG levels in rats fed HFCD + BT2 were lower than the rats fed HFCD and HFCD + BT1 in the absence of TM (Figure 4A). Conversely, HDL-C levels were increased in the HFCD + BT2 group compared to those in other groups after TM injection (Figure 4C). Interestingly, serum TC (135.1 and 120.4% compared to HFCD and HFCD + BT2, respectively) and non-HDL-C levels (143.8 and 125.8% compared to HFCD and HFCD + BT2, respectively) in rats fed HFCD + BT1 were slightly higher than other groups, which may be caused by HFCD + BT1 group containing higher levels of SFAs than HFCD groups, and a relatively higher ratio of *n*-6/*n*-3 than HFCD + BT2 group (Table 4; Figures 4B, D). However, the serum TC and non-HDL-C levels in rats fed HFCD and HFCD + BT2 had no significant differences (Figures 4B, D) despite the fact that dietary *n*-6/*n*-3 ratio of BT1 and BT2 is 0.9 and 0.4, respectively. Therefore, BT2 has a higher *n*-3 content and a lower *n*-6 content than BT1 (Table 3). Although HFCD + BT2 diet has a higher amount of SFAs than HFCD groups, HFCD + BT2 containing a lower ratio of *n*-6/*n*-3 may improve the serum lipid profiles (Table 4). The discrepancies of *n*-6/*n*-3 ratio between the diet and whole blood may be due to the innate metabolic competitiveness of *n*-6 and *n*-3 FAs. Besides, the dietary intervention did not cause significant changes in HDL-C and CRF in the absence of TM injection (Figures 4C, E). Partial replacement of dietary fat with BT1 and BT2 did not induce any changes in serum TG, TC and non-HDL-C (Figures 4A, B, D), while it decreased the CRF levels in the presence of TM (Figure 4E). These results suggest that partial replacement of dietary fat with BTs had a positive effect on CVD prevention, and BT2 diet has a positive effect on improving the serum lipid profiles.

### 3.5. Effects of partial replacement of dietary fat with BTs on fat accumulation in the liver and epididymal adipose tissue

**FIGURE 5:**
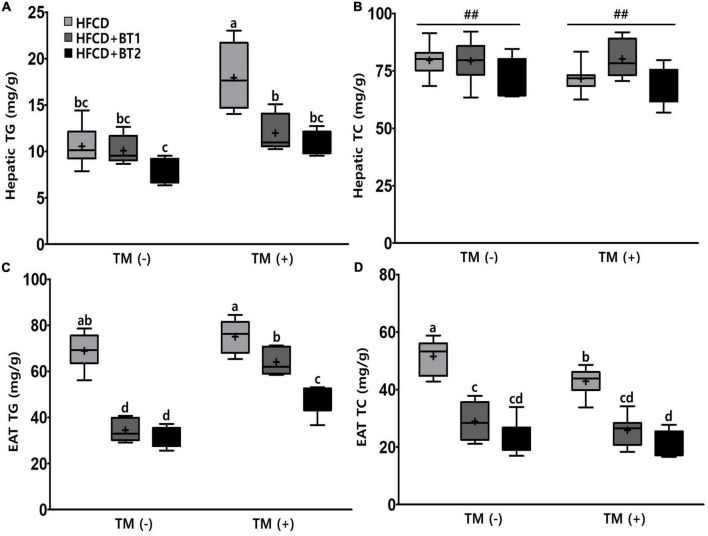
Effects of partial replacement of dietary fat with beef tallows (BTs) and tunicamycin (TM) challenge on lipid contents in the liver and epididymal adipose tissue (EAT). Five-week-old male Sprague Dawley rats were fed either a high-fat and high-cholesterol (HFCD), lard of HFCD partially replaced with regular beef tallow (HFCD + BT1), or lard of HFCD partially replaced with BT containing a lower *n*-6/*n*-3 ratio (HFCD + BT2) diets, respectively for 10 weeks, and then treated with PBS or TM (1 mg/kg). **(A)** Hepatic triglyceride (TG) levels, **(B)** hepatic total cholesterol (TC) levels, **(C)** EAT TG levels, and **(D)** EAT TC levels were measured. Values are presented as a Box-and-Whisker plots representing eight rats per group. The mean values are indicated by “+” sign. Data were analyzed using two-way ANOVA followed by Tukey’s multiple comparisons test to determine the interactions or the main effects (diet and TM stimulation). Pound indicates a significant main effect for diet (^##^*p* < 0.01). Mean values labeled with different letters indicate statistically significant difference, *p* < 0.05. HFCD, high-fat and high-cholesterol; HFCD + BT1, high-fat and high-cholesterol diet + regular beef tallow; HFCD + BT2, high-fat and high-cholesterol diet + BT containing a lower *n*-6/*n*-3 ratio; TM, tunicamycin; PBS, phosphate-buffered saline.

The serum lipid showed that the *n*-3-enhanced BTs intervention reduced the contents of serum TG but increased the HDL-C levels (Figures 4A, C), indicating *n*-3 in BT is beneficial to regulate serum lipid profiles. We also measured the liver and EAT lipid contents to determine the fat accumulation in the liver and EAT. There was no significant difference in hepatic TG contents among the three different dietary interventions in the absence of TM injection. However, hepatic TG contents were substantially increased in the rats fed with HFCD following TM injection, whereas rats given HFCD + BT1 and HFCD + BT2 significantly exhibited lower hepatic TG contents (Figure 5A). In addition, the HFCD + BT1 diet increased hepatic TC contents in rats (Figure 5B), which is consistent with previous observation of serum TC contents (Figure 4B). Moreover, the rats fed with HFCD + BT1 and HFCD + BT2, both had lower EAT TG and TC contents than those in the HFCD groups in the presence or absence of TM intervention (Figures 5C, D). These results showed that partial replacement of dietary fat with BTs could improve the lipid accumulation in the liver and adipose tissues.

### 3.6. Effects of partial replacement of dietary fat with BTs on hepatic function parameters

**FIGURE 6:**
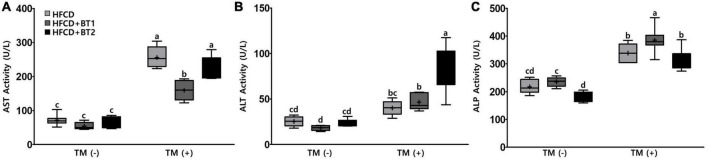
Effects of partial replacement of dietary fat with beef tallows (BTs) and tunicamycin (TM) challenge on hepatic function parameters in serum. Five-week-old male Sprague Dawley rats were fed either a high-fat and high-cholesterol (HFCD), lard of HFCD partially replaced with regular beef tallow (HFCD + BT1), or lard of HFCD partially replaced with BT containing a lower *n*-6/*n*-3 ratio (HFCD + BT2) diets, respectively for 10 weeks, and then treated with PBS or TM (1 mg/kg). **(A)** Aspartate aminotransferase (AST) activity, **(B)** alanine aminotransferase (ALT) activity, and **(C)** alkaline phosphate (ALP) activity was measured. Values are presented as a Box-and-Whisker plots representing 8 rats per group. The mean values are indicated by “+” sign. Data were analyzed using two-way ANOVA followed by Tukey’s multiple comparisons test to determine the interactions or the main effects (diet and TM stimulation). Mean values labeled with different letters indicate statistically significant difference, *p* < 0.05. HFCD, high-fat and high-cholesterol; HFCD + BT1, high-fat and high-cholesterol diet + regular beef tallow; HFCD + BT2, high-fat and high-cholesterol diet + BT containing a lower ratio of *n*-6/*n*-3 PUFA; TM, tunicamycin; PBS, phosphate-buffered saline.

Since partial replacement of dietary fat with BTs reduced hepatic TG levels after TM injection (Figure 5A), we reasoned that reducing TG levels in liver might alleviate liver function. The accumulation of TG in liver is well known to be detrimental (48). To this end, the hepatic enzyme function was assessed by examining serum ALT, AST, and ALP activities following the TM challenge. The dietary intervention did not cause any changes in AST and ALT levels, while the HFCD + BT2 diet reduced ALP levels compared to HFCD and HFCD + BT1 groups before TM injection (Figure 6). TM administration significantly increased the AST and ALP levels of HFCD + BT1 and HFCD + BT2 compared to the PBS group (Figures 6A, C). In addition, rats fed HFCD showed no significant changes in ALT levels after TM injection, whereas the HFCD + BT2 groups showed significant increased ALT levels (Figure 6B). The rats fed with HFCD + BT1 showed lower AST levels than HFCD and HFCD + BT2 group after TM injection (Figure 6A). In contrast, the ALP levels in HFCD + BT1 group higher than those in other groups, which probably is related to the higher SFAs content in HFCD + BT1 diet and higher ratio of *n*-6/*n*-3 PUFA than HFCD + BT2 diet as previously mentioned (Table 3; Figure 6C). Moreover, TM injection significantly increased ALT levels in the HFCD + BT2 group (Figure 6B). These results showed that partial replacement of lard in HFCD with *n*-3-enhanced BT may have adverse effects on hepatic function parameters.

### 3.7. Effects of partial replacement of dietary fat with BTs and TM challenge on protein expression of ER stress in liver and in epididymal adipose tissue

**FIGURE 7:**
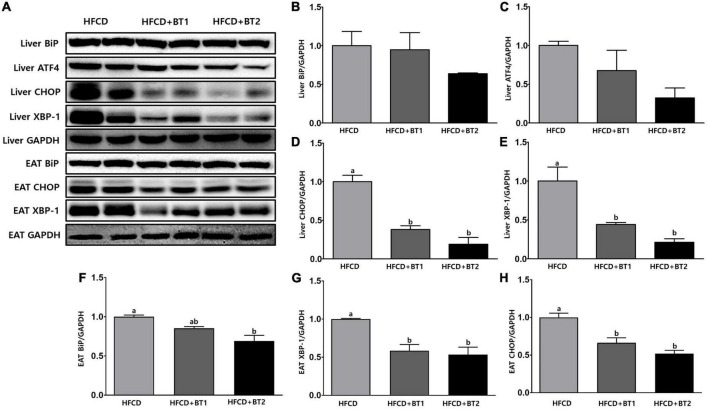
Effects of partial replacement of dietary fat with beef tallows (BTs) and tunicamycin (TM) challenge on protein expression of ER stress in liver and epididymal adipose tissue (EAT). Five-week-old male Sprague Dawley rats were fed either a high-fat and high-cholesterol (HFCD), lard of HFCD partially replaced with regular beef tallow (HFCD + BT1), or lard of HFCD partially replaced with BT containing a lower *n*-6/*n*-3 ratio (HFCD + BT2) diets, respectively for 10 weeks, and then treated with PBS or TM (1 mg/kg). **(A)** Representative western blot images, **(B)** liver Binding immunoglobulin protein (BiP) levels, **(C)** liver Activating transcription factor 4 (ATF4) levels, **(D)** liver C/EBP homologous protein (CHOP) levels, **(E)** liver X-box binding protein 1 (XBP-1) levels, **(F)** EAT BiP levels, **(G)** EAT XBP-1 levels, and **(H)** EAT CHOP levels were measured. The expression of each protein was normalized to a value for GAPDH, the internal control of protein content. Values are presented as means ± SD; *n* = 8 per individual group. Data were analyzed using one-way ANOVA followed by Tukey’s multiple comparisons test. Mean values labeled with different letters indicate statistically significant difference, *p* < 0.05. HFCD, high-fat and high-cholesterol; HFCD + BT1, high-fat and high-cholesterol diet + regular beef tallow; HFCD + BT2, high-fat and high-cholesterol diet + BT containing a lower *n*-6/*n*-3 ratio; ER stress, endoplasmic reticulum Stress; TM, tunicamycin; PBS, phosphate-buffered saline.

To understand the effects of partial replacement of dietary fat with BTs on ER stress in TM-induced SD rats, the expression of UPRs was analyzed. TM is a glycosylation inhibitor that inhibits N-linked glycosylation of nascent proteins, leading to the accumulation of unfolded proteins in the ER, inducing ER stress (47, 49). In the liver, the protein expression of BiP, ATF4, CHOP, and XBP-1 in rats fed HFCD + BT1 decreased by 0. 05-, 0. 48-, 1. 62-, and 1.26-fold, respectively compared to the HFCD group, respectively: the difference in expression of BiP and ATF4 was not statistically varied (Figures 7A–E). Compared with HFCD group, the expression of BiP, ATF4, CHOP, and XBP-1 were significantly lower in HFCD + BT2 group by, 0. 56-, 2. 08-, 4. 25-, and 3.68-fold, respectively (Figure 7); however, there was also no significant differences in the protein expression of BiP and ATF4 (Figures 7B, C). These results showed that partial replacement of dietary fat with BTs reduced the TM-induced hepatic ER stress protein expression. Therefore, we measured the expression of ER stress-related protein in EAT in TM-injected SD rats. The protein expression of CHOP and XBP-1 in rats fed HFCD + BT1 significantly decreased by 0.71- and 0.51-fold, respectively compared to the HFCD group (Figures 7A, G, H). By contrast, the protein expression of BiP in HFCD + BT1 group was not significantly different from the HFCD group (Figure 7F). Moreover, the ER stress-related proteins BiP, CHOP, and XBP-1 expression in rats fed HFCD + BT2 was, respectively lower than HFCD group by 0. 45-, 0. 87-, and 0.92-fold (Figures 7A, F–H). These results showed that partial replacement of dietary fat with BTs reduced the TM-induced ER stress protein expression in EAT.

## 4. Discussion

In this study we aimed to investigate partial replacement of lard in HFCD with *n*-3 enriched-BTs which may attenuate the risk factors of MetS and TM-induced ER stress. To induce a rodent model to mimic clinical MetS, 5-week-old male SD rats were fed HFCD, HFCD + BT1, or HFCD + BT2 diets for 10 weeks, after which they were injected with either PBS or TM. HFCD was fed to the rats as an experimental diet due to a greater negative impact on serum lipid metabolism and liver function than high fat diet (HFD) feeding (50). The composition of the dietary fats in the BT groups shows higher SFAs content, compared to the HFCD group, while BT fed groups have higher *n*-3 content and lower ratio of *n*-6/*n*-3 (Table 3). Our research question was to elucidate which was the more important factor: the amount of *n*-3 PUFA consumption or the lower ratio of *n*-6/*n*-3 PUFA in the dietary fats, to manage MetS in dietary-induced obese MetS rat model.

Our previous study demonstrated that partial replacement of dietary fat with krill oil or coconut oil improved dyslipidemia in lipopolysaccharide (LPS)-injected rats (35). We also demonstrated that replacing dietary fat with *perilla* oil or corn oil attenuates LPS-induced hepatic inflammation in rats (36). These results led us to this follow-up study to investigate the effects of partial replacement of dietary fat with PUFAs on metabolic complications. Increased levels of serum *n*-6 elevated the risk of MetS, while decreased ratio of *n*-6/*n*-3 may be effective in reducing the prevalence of IR and MetS (51), and reducing ratio of *n*-6/*n*-3 in the diet attenuates SFAs-induced weight gain in experimental rats (52). These studies suggest that diets containing high levels of *n*-6 may have adverse health effects but lowering *n*-6/*n*-3 ratio may be an effective and reasonable strategy. Moreover, the above study explored the effects of dietary intervention containing higher *n*-3, and lower *n*-6/*n*-3 ratio in rats being fed diets with high levels of SFAs. In this study, partial replacement of lard in the experimental diet with BTs caused no significant changes in body weight gain, daily food intake, daily energy intake, and FER compared to the HFCD group (Figures 1B–E), which is consistent with results from our previous studies in rats (36). Interestingly, the HFCD + BT1 group had significantly higher BW than HFCD and HFCD + BT2 groups after 4 weeks of dietary intervention (Figure 1A), which was not sustained by the end of the dietary intervention. HFCD + BT1 group had lower absolute *n*-3 content and higher ratio of *n*-6/*n*-3 than HFCD + BT2 group (Table 3), resulting in body weight changes during the dietary intervention. It has been reported that *n*-3 consumption could decrease lipid levels and glycemic factors including HOMA-IR in patients (53, 54), which is consistent with our current results (Figure 2), and our previous findings (33, 36). It has been reported that HFCD may cause the fat accumulation in the liver and adipose tissue (55), thus the weight of liver and adipose tissues were observed and we found no significant effects of dietary intervention with or without TM injection (Figure 3). However, TM injection increased the weight of RAT and PAT in the HFCD + BT2 group, and resulted in elevated weights of total WAT (Figures 3B, E, F). Albeit, the molecular mechanisms underlying the observed anabolic responses should be scrutinized in future studies. Therefore, we logically postulate that partial replacement of dietary fat with BTs may have protective effects against MetS, and further studies are need to observe the serum adiposity levels, to investigate the changes on MetS indicators.

It has been shown that *n*-3 PUFAs consumption reduces the risk of CVDs and decreases the MetS risk factors, by modulating blood lipid levels (56–59). There is a report that the intake of PUFAs with lower ratio of *n*-6/*n*-3 ameliorates hepatic steatosis and glucose metabolism (60). Therefore, the higher contents of SFAs in BT supplement may be a major risk factor for onset or progression of MetS. Decreased HDL-C, together with increased low-density lipoprotein (LDL-C) particles and TG-rich lipoproteins (TRLs) are the main components of dyslipidemia closely associated with the MetS (61). HFCD + BT2 group showed lower serum TG levels in animals without TM injection, and interestingly, the HFCD + BT1 group showed increased TC and non-HDL-C levels (Figures 4A, B, D) indicating that excessive SFA consumption may lead to dyslipidemia regardless *n*-3 PUFA contents. Injection of TM resulted in a higher HDL level in the HFCD + BT2 group. Whereas, HFCD + BT1 and HFCD + BT2 groups, respectively showed decreased CRF levels compared to the HFCD group (Figures 4C, E). These results may suggest that partial replacement of dietary fat with BTs may improve dyslipidemia, and consequently reduce CRFs caused by HFCD.

Prolonged elevated ER stress is a strong risk factor in the pathogenesis of MetS, T2DM, CVDs and obesity (23). In this study, the dietary intervention did not significantly influence ER stress related protein expression in the PBS-injected rats (data not shown). We examined the regulation of hepatic and WAT UPRs in TM-injected SD rats. Hepatocytes are ER-rich, and ER stress plays an essential role in mediating various hepatic pathological changes (62). The results showed that HFCD + BT1 and HFCD + BT2 diets significantly reduced the expression of hepatic UPR-related proteins, such as ATF4, CHOP and XBP-1 (Figures 7A, C, D, E); HFCD + BT2 diet further reduced the expression of the liver ER chaperone protein, BiP (Figures 7A, B). Obesity and some metabolic syndromes are often associated with WAT dysfunction (63), and ER stress in the WAT has a critical pathophysiological role systemically as well as in local tissues (64). In our study, replacement fatty acids (BT1 and BT2) significantly attenuated the expression of CHOP and XBP-1 proteins in the EAT (Figures 7A, G, H), while HFCD + BT2 diet remarkably reduced BiP expression in EAT (Figures 7A, F). These results support the hypothesis that *n*-3 consumption mitigates ER stress in the liver (65) and the WAT with reducing oxidative stress (66). Our previous study also reported that partial replacement of dietary fat in HFD with BTs reduced the expression of ER-related proteins in both liver and EAT (33). Partial replacement of dietary fat with BTs attenuated ER stress in the liver and EAT, while the HFCD + BT2 group containing a lower *n*-6/*n*-3 PUFA ratio seemed to be more effective in attenuating ER stress. Therefore, we logically reason that *n*-3 consumption and low ratio of *n*-6/*n*-3 PUFA may help to regulate metabolic complications, and attenuate ER stress.

There is significant debate about whether a high amount of dietary *n*-3 PUFA consumption is safe, since we observed that replacement with lower *n*-6/*n*-3 PUFA (HFCD + BT2) significantly elevated serum AST and ALT levels (Figures 6A, B). *n*-3 PUFA consumption has numerous beneficial effects; however, due to the structural instability, extra antioxidant supplementation may be necessary (58). A previous study has reported that α-tocopherol supplementation as an antioxidant ameliorates the DNA damage in human lymphocytes caused by *n*-3 (67). The AHA has reported that α-tocopherol has antioxidant and pro-oxidant properties, and that the pro-oxidant properties can be reduced by consumption of ascorbic acid (68). Moreover, a triple antioxidant combination with ascorbic acid, glutathione, and α-tocopherol has been shown to improve cholesterol levels in diabetic rats (69). Given the higher AST and ALT levels in the HFCD + BT2 group, we assume that appropriate supplementation with antioxidative reagents concomitant with *n*-3 consumption may ameliorate the hepatotoxic effects.

Partial replacement of lard in HFCD with BTs improved insulin sensitivity and lipid panels, and lowered fat accumulation in the liver and EAT in TM-injected rats. Furthermore, HFCD + BT1 and HFCD + BT2 diets attenuated the expression of TM-induced ER stress proteins in the liver and EAT. We suggest that the increasing dietary *n*-3 intake and decreasing the ratio of *n*-6/*n*-3 may alleviate HFCD-induced dyslipidemia and MetS, although HFCD + BT1 and HFCD + BT2 have shown higher SFAs levels than the HFCD group. The results of this study may serve as a basis for future clinical trials; long-term studies are needed to thoroughly confirm the feasibility of BT with higher amount of *n*-3, and lower *n*-6/*n*-3 ratio as a dietary supplement. Moreover, it would be interesting to investigate the co-intake of *n*-3 and antioxidants, such as, α-tocopherol, ascorbic acid or glutathione using *in vitro* and *in vivo* experiments to find more effective methods of *n*-3 intake (70).

## Data availability statement

The original contributions presented in the study are included in the article/supplementary material, further inquiries can be directed to the corresponding authors.

## Ethics statement

The animal study was reviewed and approved by the Institutional Animal Care Use Committee of Dankook University.

## Author contributions

JB, DY, and J-HH designed the study. JZ and JL performed data management and data analysis. JZ and JB wrote the first draft of the manuscript. All authors contributed to manuscript revision and read and approved the submitted version.
